# Sand casting safety assessment for foundry enterprises: fault tree analysis, Heinrich accident triangle, HAZOP–LOPA, bow tie model

**DOI:** 10.1098/rsos.180915

**Published:** 2018-10-24

**Authors:** Qingwei Xu, Kaili Xu, Xiwen Yao, Jinjia Zhang, Ben Wang

**Affiliations:** Key Laboratory of Ministry of Education on Safe Mining of Deep Metal Mines, School of Resources and Civil Engineering, Northeastern University, Shenyang 110819, People's Republic of China

**Keywords:** sand casting, explosion accident, fault tree analysis, Heinrich accident triangle, HAZOP–LOPA, bow tie model

## Abstract

Sand casting operations, though commonplace, pose a significant threat of explosion accidents. This paper presents a novel sand casting safety assessment technique based on fault tree analysis, Heinrich accident triangle, hazard and operability–layer of protection analysis (HAZOP–LOPA) and bow tie model components. Minimal cut sets and minimal path sets are first determined based on fault tree analysis, then the frequency of sand casting explosion accidents is calculated based on the Heinrich accident triangle. Third, the risk level of venting quality can be reduced by adopting HAZOP–LOPA; the residual risk level of venting quality remains excessive even after adopting two independent protective layers. The bow tie model is then adopted to determine the causes and consequences of venting quality. Five preventative measures are imposed to enhance the venting quality of foundry sand accompanied by 16 mitigative safety measures. Our results indicate that the risk attributable to low foundry sand venting quality can be minimized via bow tie analysis.

## 1. Introduction

Casting is a metal hot working process for producing components via mechanical manufacturing which plays an important role in the national economy [1,2]. Sand casting is the most common casting method at nearly 90% of today's foundry industry [3]. Sand casting operations threaten air pollution [4–6] and accidents [7,8], including very serious explosions. A severe sand casting explosion accident in the Anshan Iron and Steel Group foundry in China in 2012, for example, killed 13 workers and injured another 17 [7,9]. There is an urgent demand for effective safety measures to prevent sand casting explosions; such measures are predicated on highly accurate safety assessments.

Fault tree analysis is a popular safety assessment method [10–12] which involves drawing a diagram of the logical relationship between an accident in the system and its various causes. A fault tree may reveal the main causes of an accident and provide a basis for determining safety measures by subsequent qualitative and quantitative analysis of the tree. Fault tree analysis has been applied to explosion assessments in oil storage tanks [13], methane operations [14,15], air pollution-related fields [16] and traffic accidents [17–19]. The present study marks the first time that fault tree analysis has been introduced into sand casting safety assessment.

Although fault tree analysis may reveal the main causes of an explosion accident, it neither directly provides corresponding safety measures nor allows for fully comprehensive exploitation of the results in terms of safe operations. Other methods must be introduced to conduct an in-depth analysis of the causes of an explosion and eliminate any hidden dangers in operations. To determine which safety measures most effectively control critical safety hazards, hazard and operability–layer of protection analysis (HAZOP–LOPA) can be used to determine process parameters in the system that are most closely related to accidents; HAZOP–LOPA can also be used to define the causes, risk levels and consequences of accidents as identified by independent protective layers [20–22]. HAZOP–LOPA has been applied for safety assessment in various systems [23,24].

The frequency of sand casting explosion accidents must be determined in order to identify the risk level in the system. There has been only one sand casting explosion accident since 2001 according to the Ministry of Emergency Management of the People's Republic of China: the aforementioned accident at the Anshan Iron and Steel Group [7]. In this study, we calculated sand casting explosion frequency based on the Heinrich accident triangle to resolve this information deficiency [25–27].

If the hidden dangers in production are not completely eliminated after applying HAZOP–LOPA to the root causes of the explosion, further measures must be taken to more comprehensively identify critical ‘bottom events’ (i.e. root causes of an accident). The bow tie model integrates the basic causes, possible effects and corresponding safety measures related to a given accident in a transparent diagram [28–30]. The bow tie model has been applied to safety assessment in natural gas pipelines [31,32] and ore mines as well [28,33].

The main purpose of this study was to create a new approach to sand casting explosion prevention. We propose a composite safety assessment method based on fault tree analysis, Heinrich accident triangle, HAZOP–LOPA and bow tie model components. Critical bottom events of sand casting explosions are identified based on fault tree analysis; explosion frequency is then calculated by Heinrich accident triangle. The risk level of critical bottom events is then minimized by HAZOP–LOPA and the bow tie model.

## 2. Material and methods

### 2.1. Framework of the proposed method

**Figure 1.**
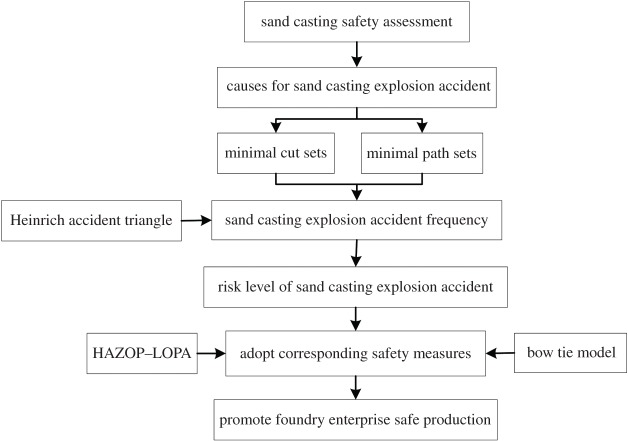
Framework of proposed composite safety assessment method.

A flow chart describing the framework of the proposed method is shown in figure 1. As shown in figure 1, to assess the sand casting, it is first necessary to determine the causes of a sand casting explosion accident based on fault tree analysis [10–12]. The roles of the minimal cut set and minimal path set are particularly important in this step. The sand casting explosion accident frequency can then be determined based on the Heinrich accident triangle [25–27]. Next, the risk level of a sand casting explosion accident can be reduced by HAZOP–LOPA [20–22] and bow tie analysis [28–30]. Corresponding safety measures can be adopted based on this information to safeguard the foundry enterprise production process.

### 2.2. Fault tree analysis

Fault tree analysis is a graphical deduction method which involves logical reasoning of ‘top’ and ‘bottom’ events under certain conditions [10–12]. It can facilitate in-depth analysis of specific accidents per the internal relationships among events in the system. It also reveals logical consistency between unit failures and system accidents according to ‘weak links’ in the system.

### 2.3. HAZOP–LOPA

HAZOP is a systematic examination method that is already commonly used in the engineering field. It can be used to assess the risk caused by mis-operation or mechanical failures in individual pieces of equipment as well as an entire system [23,34,35]. HAZOP is operated by using keywords as a guide to identify deviations in process parameters in the system, and then by analysing the causes and effects of such deviations to determine which measures should be taken to optimize (e.g. safeguard) the system.

LOPA is a systematic method for assessing the effectiveness of protective layers and for making risk decisions by qualitative risk analysis [24,36,37]. Its main purpose is to ensure that there are sufficient protective layers to ensure the foundry enterprise does not contain excessive risk. LOPA is a semi-quantitative accident scenario assessment method that reflects the causes, effects and independent protective layers related to an accident.

HAZOP–LOPA reveals any deviations in system process parameters which may result in an accident; it allows the user to define causes, risk levels and consequences to prevent accidents via independent protective layers [20–22].

### 2.4. Bow tie model

**Figure 2.**
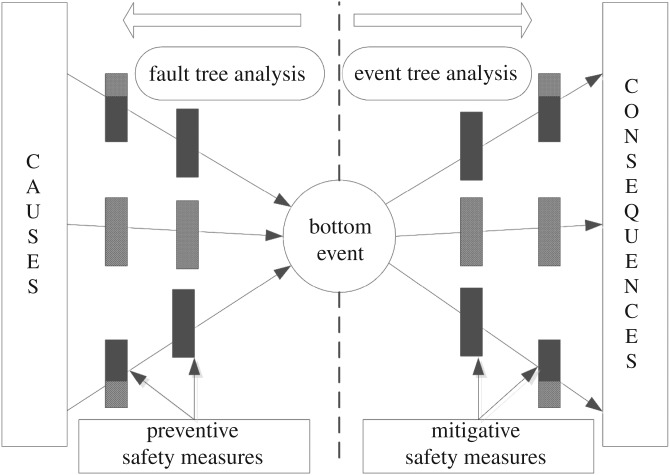
Bow tie model flow chart.

The bow tie model is characterized by a fault tree on its left-hand side and event tree on its right-hand side with a ‘bottom event’ in its centre (figure 2) [28–30]. The basic causes of the bottom event may result in accidents indicated on the left side of the bow tie. The fatal consequences of the bottom event (e.g. property loss and casualties) are indicated on the right side of the bow tie. Preventive safety measures are set on the left side (fault tree) of the bottom event and mitigative safety measures are set on the right side (event tree) of the bottom event.

## 3. Results

**Figure 3.**
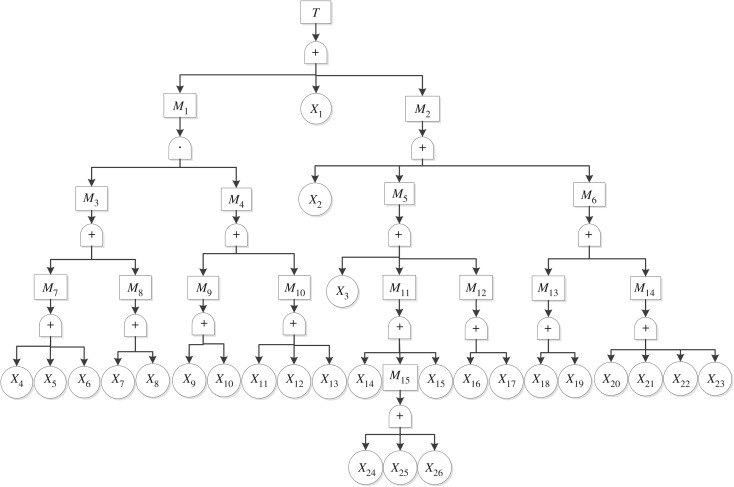
Sand casting explosion accident fault tree.

The fault tree of a sand casting explosion accident is shown in figure 3. In figure 3, the sign ‘·’ indicates an ‘AND’ gate corresponding to a logic relation in which the output event occurs only if all of the input events exist simultaneously. The sign ‘+’ marks an ‘OR’ gate corresponding to a logic relation in which the output event occurs if any of the input events occur. Relatively innocuous sand casting explosion conditions are considered here, such as boiling, wherein molten metal splashes occur due to poor exhaust properties in the sand mould. Other symbols included in the fault tree shown in figure 3 are defined in table 1.

**Table 1.** Fault tree symbol legend.

symbol	description
*T*	sand casting explosion accident
*M*_1_	molten metal meets ponding
*M*_2_	cavity exhaust blocked
*M*_3_	ponding anomaly
*M*_4_	sand casting with ponding anomaly
*M*_5_	large gas evolution of foundry sand
*M*_6_	gas channel abnormal
*M*_7_	surface water inflowing
*M*_8_	ground water permeated
*M*_9_	no ponding detection
*M*_10_	ponding detection failure
*M*_11_	high moisture content
*M*_12_	large gas evolution of additive
*M*_13_	riser abnormal
*M*_14_	gas vent abnormal
*M*_15_	insufficient drying
*X*_1_	low sand strength
*X*_2_	low sand venting quality
*X*_3_	large compactability
*X*_4_	water dam does not protrude from the ground
*X*_5_	factory leak
*X*_6_	other operations introduce water leaks
*X*_7_	no water dam
*X*_8_	water dam failure
*X*_9_	no monitoring equipment
*X*_10_	monitoring equipment not in use
*X*_11_	excessive alarm value
*X*_12_	monitoring device damaged
*X*_13_	monitoring points unevenly arranged
*X*_14_	high water content in sand mixing process
*X*_15_	not moulded immediately after drying
*X*_16_	unreasonable additive configuration
*X*_17_	use inferior additives
*X*_18_	no riser
*X*_19_	unreasonable riser position
*X*_20_	no gas vent
*X*_21_	gas vent aperture too small
*X*_22_	insufficient gas vent depth
*X*_23_	unreasonable gas vent position
*X*_24_	no drying
*X*_25_	insufficient drying
*X*_26_	short drying time

The fault tree structure function of a sand casting explosion is as follows, based on figure 3:

3.1 $$T=M_{1}+X_{1}+M_{2}.$$

There are altogether 41 minimal cut sets of the sand casting explosion accident after simplification by Boolean algebra: {*X*_1_}, {*X*_2_}, {*X*_3_}, {*X*_14_}, {*X*_15_}, {*X*_16_}, {*X*_17_}, {*X*_18_}, {*X*_19_}, {*X*_20_}, {*X*_21_}, {*X*_22_}, {*X*_23_}, {*X*_24_}, {*X*_25_}, {*X*_26_}, {*X*_4_, *X*_9_}, {*X*_4_, *X*_10_}, {*X*_4_, *X*_11_}, {*X*_4_, *X*_12_}, {*X*_4_, *X*_13_}, {*X*_5_, *X*_9_}, {*X*_5_, *X*_10_}, {*X*_5_, *X*_11_}, {*X*_5_, *X*_12_}, {*X*_5_, *X*_13_}, {*X*_6_, *X*_9_}, {*X*_6_, *X*_10_}, {*X*_6_, *X*_11_}, {*X*_6_, *X*_12_}, {*X*_6_, *X*_13_}, {*X*_7_, *X*_9_}, {*X*_7_, *X*_10_}, {*X*_7_, *X*_11_}, {*X*_7_, *X*_12_}, {*X*_7_, *X*_13_}, {*X*_8_, *X*_9_}, {*X*_8_, *X*_10_}, {*X*_8_, *X*_11_}, {*X*_8_, *X*_12_}, {*X*_8_, *X*_13_}.

The minimal path sets of the sand casting explosion accident can be obtained similarly: {*X*_1_, *X*_2_, *X*_3_, *X*_4_, *X*_5_, *X*_6_, *X*_7_, *X*_8_, *X*_14_, *X*_15_, *X*_16_, *X*_17_, *X*_18_, *X*_19_, *X*_20_, *X*_21_, *X*_22_, *X*_23_, *X*_24_, *X*_25_, *X*_26_}, {*X*_1_, *X*_2_, *X*_3_, *X*_9_, *X*_10_, *X*_11_, *X*_12_, *X*_13_, *X*_14_, *X*_15_, *X*_16_, *X*_17_, *X*_18_, *X*_19_, *X*_20_, *X*_21_, *X*_22_, *X*_23_, *X*_24_, *X*_25_, *X*_26_}.

## 4. Discussion

### 4.1. Minimal cut set and minimal path set roles

The minimal cut set plays an important role in fault tree analysis. First, it indicates the risk in the system. There are 41 minimal cut sets in the sand casting explosion fault tree, indicating that an explosion may have 41 different causes; in short, that sand casting is very dangerous. Second, it indicates the causes of an explosion. The explosion must be caused by the simultaneous occurrence of bottom events in at least one minimal cut set. The causes of an explosion can be readily identified once the explosion has occurred. Fault tree analysis also reveals directional control and prevention measures which reduce the risk of explosions. It clearly indicates potential opportunities to reduce the occurrence of such accidents.

The minimal path set plays a similar role to the minimal cut set in fault tree analysis. First, it indicates the safety of the system. Sand casting explosions can be avoided by preventing all the bottom events in a random minimal path set. The preferred plan to ensure system safety can also be achieved according to the minimal path set. Each minimal path set is a solid plan to prevent explosions; the preferred plan should also be determined as per the necessary technology, time and money.

Li & Ji [7] studied the amount of water needed for a sand casting explosion. In an interesting departure from the extant literature, we focus here on the causes of sand casting explosions. According to Li & Ji, a sand casting explosion can be prevented by reducing the moisture content of the sand mould; in this study, we found that a sand casting explosion can be prevented by adopting corresponding safety measures. We also provide safety measures which may guarantee safe production in the foundry enterprise from several other perspectives.

### 4.2. Sand casting explosion accident frequency based on Heinrich accident triangle

**Figure 4.**
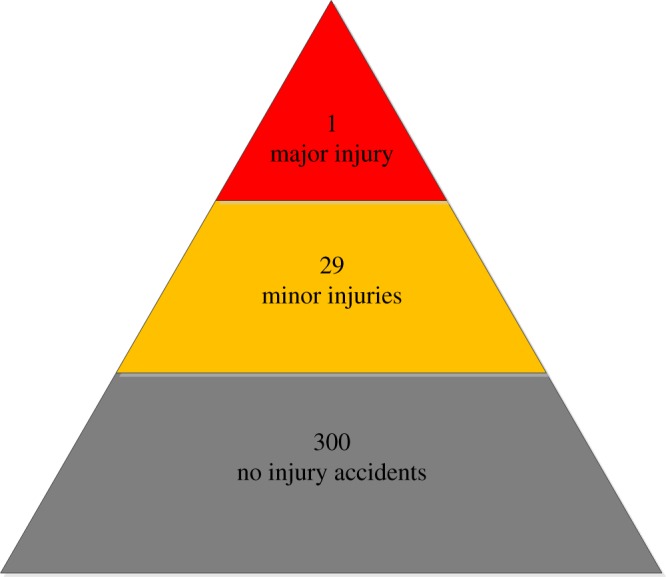
Heinrich accident triangle.

There has been only one sand casting explosion accident since 2001 according to the Ministry of Emergency Management of the People's Republic of China (the accident at the Anshan Iron and Steel Group [7], as mentioned above). According to our Heinrich accident triangle, there are 29 minor-injury and 300 no-injury accidents hidden behind one major injury (figure 4) [25–27].

The Heinrich triangle encompasses 550 000 mechanical accidents. Fatal and major injuries account for 1666 of the total, minor injuries for 48 334 and non-injuries for the remainder. According to Heinrich, the ratio of major (including fatal) injuries, minor injuries and non-injuries is 1 : 29 : 300.

The casting process, as discussed above, is an important component of mechanical engineering. Sand casting explosions can be easily placed onto a Heinrich accident triangle. Here, our count period is from 2001 to 2012 for about 24 000 foundry enterprises in China. The frequency of sand casting explosions in this sample is 1.25 × 10^−3^ per annum.

### 4.3. Risk level of sand casting explosion accident

The frequency level of a sand casting explosion accident is ‘probable’ according to table 2.

**Table 2.** Accident frequency level [38].

level	frequency (annual)	possibility
1	less than 10^−6^	eliminated
2	10^−6^–10^−5^	improbable
3	10^−5^–10^−4^	remote
4	10^−4^–10^−3^	occasional
5	10^−3^–10^−2^	probable
6	10^−2^–10^−1^	frequent
7	greater than 10^−1^	very frequent

The severity of accident consequences (table 3) must also be determined to define the explosion risk level in the whole system.

**Table 3.** Accident consequence severity level.

level	severity	consequence
1	negligible	simple medical treatment without hospitalization or brief malaise
2	minor	restricted work or slight restriction due to injury
3	medium	grievous injury or occupational disease
4	major	one or two fatalities or disablements, or three to nine serious injuries
5	catastrophic	three or more deaths, or ten or more serious injuries

A sand casting explosion accident can cause up to 13 deaths and 17 injuries [7] which is defined by the ‘catastrophic’ severity level (table 3). Let the accident frequency be *F* and the severity of accident consequences be *S*, then the accident risk level can be calculated as follows [38]:

4.1 $$R_{l}=F\times S.$$

The risk ranking matrix can be found in the electronic supplementary material. In our case, the risk level of sand casting explosions is 25; this value represents the frequency of accidents and the severity of their consequences. Safety measures must be adopted immediately based on the rubric given in table 4.

**Table 4.** Risk level and required measures [38].

risk level	required measures
less than 6	no safety measures required
7–12	safety measures should be adopted as conditions allow
13–20	safety measures should be adopted designedly
greater than 20	safety measures must be adopted immediately

### 4.4. HAZOP–LOPA of venting quality

The consequences of explosions created where the molten metal meets the ponding are of utmost seriousness in the sand casting process. The volume of the ponding expands rapidly upon meeting the molten metal. This can cause a large amount of energy to be trapped due to cavity exhaust blockage leading to an explosion. Routine inspection of any abnormal ponding is crucial to this effect. There are many factors that influence cavity exhaust which are difficult to troubleshoot, which results in a relatively high probability of cavity exhaust blockage. Here, we focus specifically on the cavity exhaust in regard to casting safety.

The risk level of sand casting explosions is 25 according to the Heinrich accident triangle, indicating that safety measures must be adopted immediately. Molten metal is very likely to contact the ponding and result in an explosion if the cavity exhaust is blocked. Venting quality is one of the most important factors affecting the cavity exhaust. The venting quality of foundry sand refers to the ability of sand to allow gas to pass through after compaction. Low venting quality can lead to cavity exhaust blockage. Therefore, we ran a HAZOP–LOPA [20–22] on venting quality in an effort to reduce the risk of explosions as shown in table 5.

**Table 5.** HAZOP–LOPA of venting quality.

guide word	element	cause	consequence	risk level	independent protective layer	residual risk level
less	venting quality	1. small particle size of foundry sand	1. explosion	25	1. prediction of sand casting explosion	10–15
		2. high humidity of foundry sand	2. boiling		2. guaranteed roast time of foundry sand	

As shown in table 5, the explosion risk level caused by low venting quality remained high after adopting two independent protective layers, so we sought further safety measures to control venting quality and minimize the likelihood of explosions.

### 4.5. Bow tie analysis of venting quality

**Figure 5.**
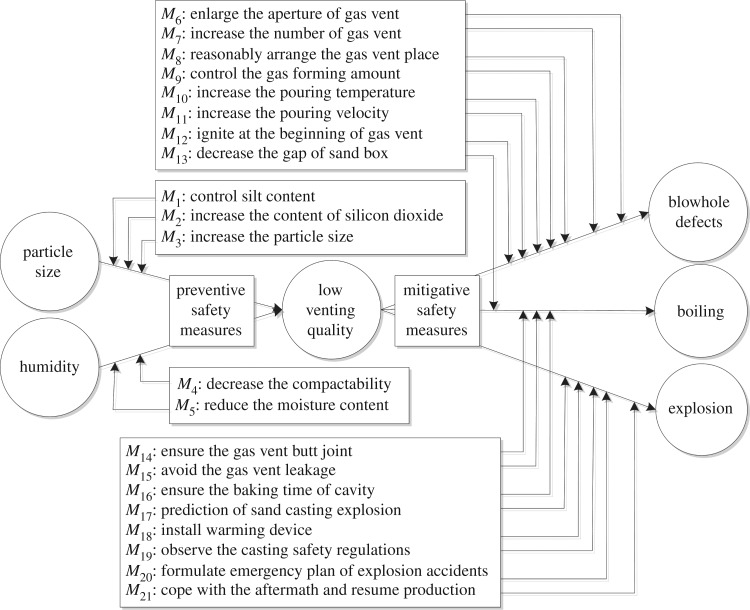
Bow tie analysis of low foundry sand venting quality.

Previous researchers [10–12] have conducted simple analyses to identify critical bottom events in safety assessments but failed to identify the causes and effects of said bottom events and thus failed to effectively enhance enterprise safety. We selected low venting quality as the critical bottom event for bow tie analysis, identified its causes and effects and defined corresponding safety measures to prevent accidents (figure 5).

Two causes that can lead to low foundry sand venting quality are imposed on the left-hand side (fault tree side) of the bow tie. Three consequences of low venting quality are set on the right-hand side (event tree side) of the bow tie, which belongs to the event tree analysis. Five corresponding preventive safety measures are set on the left side and 16 mitigative safety measures on the right. After taking 5 preventive and 16 mitigative safety measures (figure 5), the severity of a sand casting explosion accident caused by low venting quality can be reduced to minor or negligible (table 3), at which point the frequency level of sand casting explosion accidents is probable (table 2) and the residual risk level of a sand casting explosion accident is between 5 and 10. That is, the risk level of sand casting explosion accident has been reduced from 25 to 5 (the maximum residual risk level is 10) via the bow tie analysis of low foundry sand venting quality (figure 5). The results presented in figure 5 and the above analysis altogether suggest that the bow tie model can be used to reduce the risk level of low foundry sand venting quality.

Foundry sand may not be effective, however, at excessively high venting quality. When there is a large gap between sand particles, the molten metal may fall into the casting and create a rough surface or even penetrate the metal. The venting quality of foundry sand must be controlled within a suitable range.

## 5. Conclusion

This paper proposed a composite sand casting safety assessment approach based on fault tree analysis, Heinrich accident triangle, HAZOP–LOPA and bow tie model components. Our main conclusions can be summarized as follows.

We first achieved minimal cut sets and minimal path sets based on fault tree analysis resulting in 41 minimal cut sets and 2 minimal path sets of a sand casting explosion accident. We next calculated the frequency of sand casting explosions based on the Heinrich accident triangle; the result was 1.25 × 10^−3^ per annum. We attempted to control the risk level of venting quality by adopting HAZOP–LOPA, but the residual risk level of venting quality was still high after adopting two independent protective layers. Finally, we used a bow tie model to identify the causes and effects of venting quality and to define corresponding safety measures which may prevent explosions.

## Supplementary Material

Risk ranking matrix

## Data accessibility

The risk ranking matrix supporting this paper has been uploaded as the electronic supplementary material.
