# Supplementary material for: Sand casting safety assessment for foundry enterprises: fault tree analysis, Heinrich accident triangle, HAZOP–LOPA, bow tie model
Source: R Soc Open Sci. 2018 Oct 24;5(10):180915. doi: 10.1098/rsos.180915 (PMC6227990; doi:10.1098/rsos.180915)
Supplement: Risk ranking matrix [file rsos180915supp1.doc]

***Royal Society Open Science***

Sand casting safety assessment for foundry enterprises: fault tree analysis, Heinrich accident triangle, HAZOP-LOPA, bow tie model

Qingwei Xu, Kaili Xu^*^, Xiwen Yao, Jinjia Zhang and Ben Wang

Key Laboratory of Ministry of Education on Safe Mining of Deep Metal Mines, School of Resources and Civil Engineering, Northeastern University, Shenyang 110819, P. R. China

*Correspondence: [xklsafety@163.com](mailto:xklsafety@163.com)

The risk ranking matrix is shown in [Table 1](#table1).

**Table 1**. Risk ranking matrix

| Severity level  Frequency level | | Level 1 | Level 2 | Level 3 | Level 4 | Level 5 |
| --- | --- | --- | --- | --- | --- | --- |
|  |  | Negligible | Minor | Medium | Major | Catastrophic |
| Level 1 | Eliminated (<10^-6^) | 1 | 2 | 3 | 4 | 5 |
| Level 2 | Improbable (10^-6^~10^-5^) | 2 | 4 | 6 | 8 | 10 |
| Level 3 | Remote (10^-5^~10^-4^) | 3 | 6 | 9 | 12 | 15 |
| Level 4 | Occasional (10^-4^~10^-3^) | 4 | 8 | 12 | 16 | 20 |
| Level 5 | Probable (10^-3^~10^-2^) | 5 | 10 | 15 | 20 | 25 |
| Level 6 | Frequent (10^-2^~10^-1^) | 6 | 12 | 18 | 24 | 30 |
| Level 7 | Very frequent (>10^-1^) | 7 | 14 | 21 | 28 | 35 |
